# Resveratrol increases AdipoR1 and AdipoR2 expression in type 2 diabetic nephropathy

**DOI:** 10.1186/s12967-016-0922-9

**Published:** 2016-06-11

**Authors:** Hoon Suk Park, Ji Hee Lim, Min Young Kim, Yaeni Kim, You Ah Hong, Sun Ryoung Choi, Sungjin Chung, Hyung Wook Kim, Bum Soon Choi, Yong Soo Kim, Yoon Sik Chang, Cheol Whee Park

**Affiliations:** Division of Nephrology, Department of Internal Medicine, College of Medicine, The Catholic University of Korea, Seoul, Republic of Korea; Department of Internal Medicine, Seoul St. Mary’s Hospital, The Catholic University of Korea, #505, Banpo-Dong, Seocho-Ku, Seoul, 137-040 Republic of Korea

**Keywords:** Adiponectin, 5′-adenosine monophosphate-activated protein kinase, Diabetic nephropathy, Resveratrol

## Abstract

**Background:**

Adiponectin has multiple functions including insulin sensitization, anti-inflammation and antiatherogenesis in various organs. Adiponectin activates 5′-adenosine monophosphate-activated protein kinase (AMPK) and peroxisome proliferator-activated receptor (PPAR)α via the adiponectin receptor (AdipoR) 1 and 2, which are critical for regulating lipids and glucose homeostasis and for controlling oxidative stress. We investigated whether resveratrol can inhibit renal damage in type 2 diabetic *db/db* mice and the underlying mechanisms of its effects.

**Methods:**

Four groups of male C57 BLKS/J *db/m* and *db/db* mice and human glomerular endothelial cells (HGECs) were used. Resveratrol was administered to diabetic and nondiabetic mice by oral gavage for 12 weeks starting at 8 weeks of age.

**Results:**

In *db/db* mice, resveratrol increased serum adiponectin levels and decreased albuminuria, glomerular matrix expansion, inflammation and apoptosis in the glomerulus. Resveratrol increased the phosphorylation of AMPK and silent information regulator T1 (SIRT1), and decreased phosphorylation of downstream effectors class O forkhead box (FoxO)1 and FoxO3a via increasing AdipoR1 and AdipoR2 in the renal cortex. Furthermore, resveratrol increased expression of PPARγ coactivator (PGC)-1α, estrogen-related receptor-1α, and phosphorylated acetyl-CoA carboxylase and decreased sterol regulatory element-binding protein 1. This effect lowered the content of nonesterified fatty acid and triacylglycerol in the kidneys, decreasing apoptosis, oxidative stress and activating endothelial nitric oxide synthase. Resveratrol prevented cultured HGECs from undergoing high-glucose-induced oxidative stress and apoptosis by activating the AMPK–SIRT1–PGC–1α axis and PPARα through increases in AdipoR1 and AdipoR2 expression.

**Conclusions:**

These results suggest that resveratrol prevents diabetic nephropathy by ameliorating lipotoxicity, oxidative stress, apoptosis and endothelial dysfunction via increasing AdipoR1 and AdipoR2 expression.

**Electronic supplementary material:**

The online version of this article (doi:10.1186/s12967-016-0922-9) contains supplementary material, which is available to authorized users.

## Background

Diabetic nephropathy (DN) is the most common and most rapidly growing cause of end-stage renal disease. Treatment of DN by renal replacement therapy is a major expense, also in Korea [1]. Numerous factors including hyperglycemia-induced oxidative stress and inflammation contribute to the pathogenesis and progression of diabetic chronic kidney disease (CKD). Some studies have demonstrated that accumulation of lipids in the kidneys plays a crucial role in the progression of diabetic renal damage [2, 3], suggesting that lipotoxicity of free fatty acids or triglycerides and lipotoxicity-induced oxidative stress may critically contribute to the pathogenesis of diabetic CKD. However, the underlying molecular mechanisms of these effects are unclear.

Adiponectin is an adipokine secreted by adipose tissue that is downregulated in insulin-resistant obesity. Adiponectin can suppress oxidative stress and inflammation, leading to antidiabetic and antiatherosclerotic effects [4]. Adiponectin protects kidneys by reducing albuminuria, which is a reliable marker for renal dysfunction in diabetes mellitus (DM) [5, 6]. Adiponectin exerts its biological effects through adiponectin receptors (AdipoR), including AdipoR1 and AdipoR2. AdipoR1 mediates increases in 5′-adenosine monophosphate-activated protein kinase (AMPK), and AdipoR2 activates peroxisome proliferator-activated receptor (PPAR)α. AdipoR1 is widely expressed in muscle tissue, while AdipoR2 is expressed predominantly in the liver [7, 8]. Studies investigating the distribution of AdipoR1 and AdipoR2 and their function in kidneys suggested that activation of AdipoR1 and AdipoR2 could prevent and ameliorate DN, especially in type 2 DM.

Resveratrol is a natural plant polyphenol that may target aging and obesity-related chronic disease by regulating inflammation and oxidative stress. Resveratrol is an activator of AMPK, which is involved in controlling oxidative stress and in lipid and glucose homeostasis [9–12]. AMPK participates in the regulation of other cellular processes including autophagy, apoptosis, and inflammation. Previous studies demonstrated that resveratrol alleviated alcoholic fatty liver disease in mice by increasing hepatic AdipoR1/R2 expression [13] and attenuated DN by increasing renal AdipoR1 expression in rats with streptozotocin-induced diabetes [14]. In this study, we investigated whether the beneficial effects of resveratrol are associated with AdipoR1 and AdipoR2 activation in type 2 diabetic *db/db* mice.

## Methods

### Experimental methods

Six-week-old male C57BLKS/J *db/m* and *db/db* mice were purchased from Jackson Laboratories (Bar Harbor, ME, USA) and divided into four groups that received either 0.5 % carboxymethyl cellulose sodium salt (CMC) or resveratrol (Sigma-Aldrich, St. Louis, MO, USA). The resveratrol was dissolved in 0.5 % CMC and 20 mg/kg/day was administered to *db/db* Res (n = 8) and *db/m* Res (n = 8) mice for 12 weeks starting at 8 weeks of age [9]. Control *db/db* (n = 8) and *db/m* (n = 8) mice received only 0.5 % CMC. At week 20, all animals were anesthetized by intraperitoneal injection of 30 mg/kg tiletamine plus zolazepam (Zoletil; Virbac, Carros, France) and 10 mg/kg xylazine hydrochloride (Rompun; Bayer, Leuverkusen, Germany). Mice were euthanized and kidneys removed. All experiments including Western blots were performed using renal cortex samples. Kidneys were rapidly dissected and fixed in normal-buffered 10 % formalin for immunohistochemical analyses. Blood was collected from the left ventricle and plasma was stored at −70 °C.

### Ethics statement

All animal experiments were performed in accordance with the Laboratory Animals Welfare Act and the Guide for the Care and Use of Laboratory Animals, and were approved by the Institutional Animal Care and Use Committee (IACUC) at the College of Medicine, the Catholic University of Korea (CUMC-2012-0118-02). All procedures complied with the Guide for the Care and Use of Laboratory Animals (National Institutes of Health Publication No. 85–23, revised 1996).

### Measurement of serum parameters

After 12 weeks of resveratrol treatment, blood glucose was measured using an Accu-check meter (Roche Diagnostics, St. Louis, MO, USA). Hemoglobin A1c (HbA1c) was determined from red cell lysates by HPLC (Bio-Rad, Richmond, CA, USA). The concentration of serum adiponectin was determined by ELISA (Biosource, Camarillo, CA, USA). Total cholesterol and triacylglycerol (TG) concentrations were measured with an autoanalyzer (Hitachi 917, Tokyo, Japan) using commercial kits (Wako, Osaka, Japan). Nonesterified fatty acid (NEFA) levels were measured with a JCA-BM1250 automatic analyzer (JEOL, Tokyo, Japan).

### Assessment of renal function, oxidative stress and intrarenal lipids

A 24-h urine sample was obtained from mice at 20 weeks using metabolic cages, and urinary albumin concentrations were measured by immunoassay (Bayer, Elkhart, IN, USA). Plasma and urine creatinine concentrations were measured using HPLC (Beckman Instruments, Fullerton, CA, USA). To evaluate oxidative stress, we measured the 24-h urinary 8-hydroxy-2′-deoxyguanosine (8-OH-dG; OXIS Health Products, Inc., Portland, OR, USA) and 8-epi-prostaglandin F2α (8-epi-PGF2α; Oxis Research, Foster City, CA, USA) levels. Kidney lipids were extracted using the method of Bligh and Dyer with slight modifications as previously described (Waco, Osaka, Japan) [15]. Furthermore, to evaluate the effect of resveratrol on lipid accumulation in the glomerulus, we performed oil red O staining of frozen renal tissue.

### Light microscopy

Kidney samples were fixed in 10 % buffered formalin and embedded in paraffin. Histology was assessed following periodic acid–Schiff (PAS) staining. The mesangial matrix and glomerular tuft areas were quantified for each glomerular cross-section using PAS-stained sections as previously reported [16]. More than 30 glomeruli, cut through the vascular pole, were counted per kidney and the average was used for analysis.

### Immunohistochemistry and terminal deoxynucleotidyl transferase-mediated dUTP nick-end labeling (TUNEL) assay

For immunohistochemistry, 4-μm sections were deparaffinized, hydrated in ethanol, treated with an antigen-unmasking solution of 10 mmol/L sodium citrate buffer, pH 6.0, and washed with phosphate buffered saline (PBS). Sections were incubated with 3 % H_2_O_2_ in methanol to block endogenous peroxidase activity. Nonspecific binding was blocked with 10 % normal goat serum in PBS. Sections were incubated overnight with antibodies against transforming growth factor-β1 (TGF-β1) (1:100; R&D Systems, Minneapolis, MN, USA), type IV collagen (Col IV) (1:200; Biodesign International, Saco, ME, USA), F4/80 (1:50; Serotec, Oxford, UK), or 8-OH-dG (1:100; CosmoBio, Tokyo, Japan) in a humidified chamber at 4 °C. Antibody binding was visualized with peroxidase-conjugated secondary antibody using Vector Impress kits (Vector Laboratories, Burlingame, CA, USA) and 3,3-diaminobenzidine substrate solution. Sections were dehydrated in ethanol, cleared in xylene, and mounted without counterstaining. All sections were examined in a blinded manner using light microscopy (Olympus BX-50, Olympus Optical, Tokyo, Japan). Col IV expression was detected using a tyramide signal amplification fluorescence system (PerkinElmer, Waltham, MA, USA). The proportion of apoptotic cells was determined using ApopTaq In Situ Apoptosis Detection kits (Chemicon-Millipore, Billerica, MA, USA), based on the TUNEL assay. To quantify staining proportions, approximately 20 views (×400 magnification) in the renal cortex and corticomedullary junction were randomly imaged from each slide and analyzed as density × positive area/glomerular total area using a computer image analysis program (Scion Image Beta 4.0.2, Frederick, MD, USA).

### Western blots

With the total proteins from the renal cortical tissues from each groups as well as in vitro cell lines, western assay was performed with specific antibodies for AdipoR1, AdipoR2, phospho-Thr^172^ AMPK, total AMPK, silent information regulator T1(SIRT1), PPARα, phospho-Ser^256^ class O forkhead box (FoxO)1, total FoxO1, phospho-Ser^253^ FoxO3a, total FoxO3a, PPARγ coactivator (PGC)-1α, estrogen-related receptor (ERR)-1α, sterol regulatory element-binding protein (SREBP)-1c, phosphorylated acetyl-CoA carboxylase (pACC), total ACC, phospho-Ser^1177^ endothelial nitric oxide synthase (eNOS), total eNOS, B cell leukemia/lymphoma 2 (Bcl-2), Bcl-2-associated X protein (Bax) and β-actin (see the Additional file 1 for further details).

### Cell culture and small interfering RNA (siRNA) transfection

Human glomerular endothelial cells (HGECs) were purchased from Anigio-Proteomie (Boston, MA, USA) and subcultured in endo-growth media (Angio-Proteomie). The HGECs were then exposed to low glucose or high glucose, with or without the additional 6-h application of resveratrol (50 μM). siRNAs, targeted to AdipoR1 and AdipoR2 and scrambled siRNA (siRNA cont) were complexed with transfection reagent (Lipofectamin 2000; Invitrogen, Carlsbad, CA, USA), according to the manufacturer’s instructions. The proportion of apoptotic cells was determined using ApopTaq In Situ Apoptosis Detection kits, based on the TUNEL assay. To quantify staining proportions, approximately 20 views (×400 magnification) were randomly imaged from each slide (see the Additional file 1 for further details).

### Immunofluorescence analysis in the HGECs

To evaluate the effects of resveratrol on AdipoR1 and AdipoR2 expression, we performed immunofluorescence analysis for AdipoR1 (Abcam) and AdipoR2 (Abcam) in the HGECs using a tyramide signal amplification fluorescence system (PerkinElmer) and counterstained with 4,6-diamidino-2-phenylindole (DAPI).

### Statistical analysis

Data are expressed as mean ± standard deviation (SD). Differences between groups were examined for statistical significance using ANOVA with Bonferroni’s correction using SPSS version 11.5 (SPSS, Chicago, IL, USA). A p value <0.05 was considered significant.

## Results

### Physical and biochemical characteristics in mice

Body weight for the diabetic *db/db* and *db/db* Res mice was significantly higher than for nondiabetic *db/m* and *db/m* Res mice. Kidney weights did not differ between these groups. Fasting blood glucose and HbA1c were significantly higher for the *db/db* and *db/db* Res mice compared with the *db/m* and *db/m* Res mice. No differences between the groups in blood urea nitrogen and creatinine levels were observed. Albuminuria and urine volume were significantly increased in the *db/db* mice compared with the *db/m* and *db/m* Res mice. Resveratrol treatment ameliorated albuminuria and significantly decreased urine volume in *db/db* mice. In *db/m* mice, resveratrol treatment did not result in any change of the serum adiponectin level. In contrast, in *db/db* mice it slightly but significantly increased the serum adiponectin level (Table 1).

**Table 1:** Biochemical and physical characteristics of the study groups

	*Db/m* control	*Db/m* Res	*Db/db* control	*Db/db* Res
Body weight (g)	30.6 ± 1.6	29.7 ± 2.0	40.3 ± 5.3*	41.8 ± 4.3*
Kidney weight (g)	0.19 ± 0.01	0.19 ± 0.03	0.21 ± 0.03	0.22 ± 0.02
FBS (mg/dL)	169 ± 53	180 ± 35	242 ± 49*	247 ± 91*
HbA1c (%)	4.1 ± 0.1	4.1 ± 0.1	12.2 ± 1.8*	12.7 ± 1.0*
BUN (mg/dL)	0.32 ± 0.05	0.35 ± 0.04	0.36 ± 0.03	0.37 ± 0.02
Serum Cr (mg/dL)	0.079 ± 0.008	0.081 ± 0.010	0.082 ± 0.011	0.080 ± 0.009
Serum adiponectin (μg/mL)	11.6 ± 1.0	11.8 ± 0.9	4.5 ± 0.4*	6.4 ± 0.5**
Urine volume (mL)	0.7 ± 0.2	0.8 ± 0.2	8.7 ± 2.2*	3.3 ± 1.1
24-h albuminuria (μg/day)	10.0 ± 4.2	9.0 ± 1.4	140.0 ± 34.2*	30.1 ± 15.5

*Res* resveratrol, *FBS* fasting blood sugar, *HbA1c* hemoglobin A1c, *Cr* creatinine, *BUN* blood urea nitrogen

* p < 0.001 compared with other groups, ** p < 0.05 compared with *db/db* control and p < 0.01 compared with *db/m* control and *db/m* Res

### Effects of resveratrol on renal phenotypes, TGF-β1, Col IV and F4/80

**Fig. 1:**
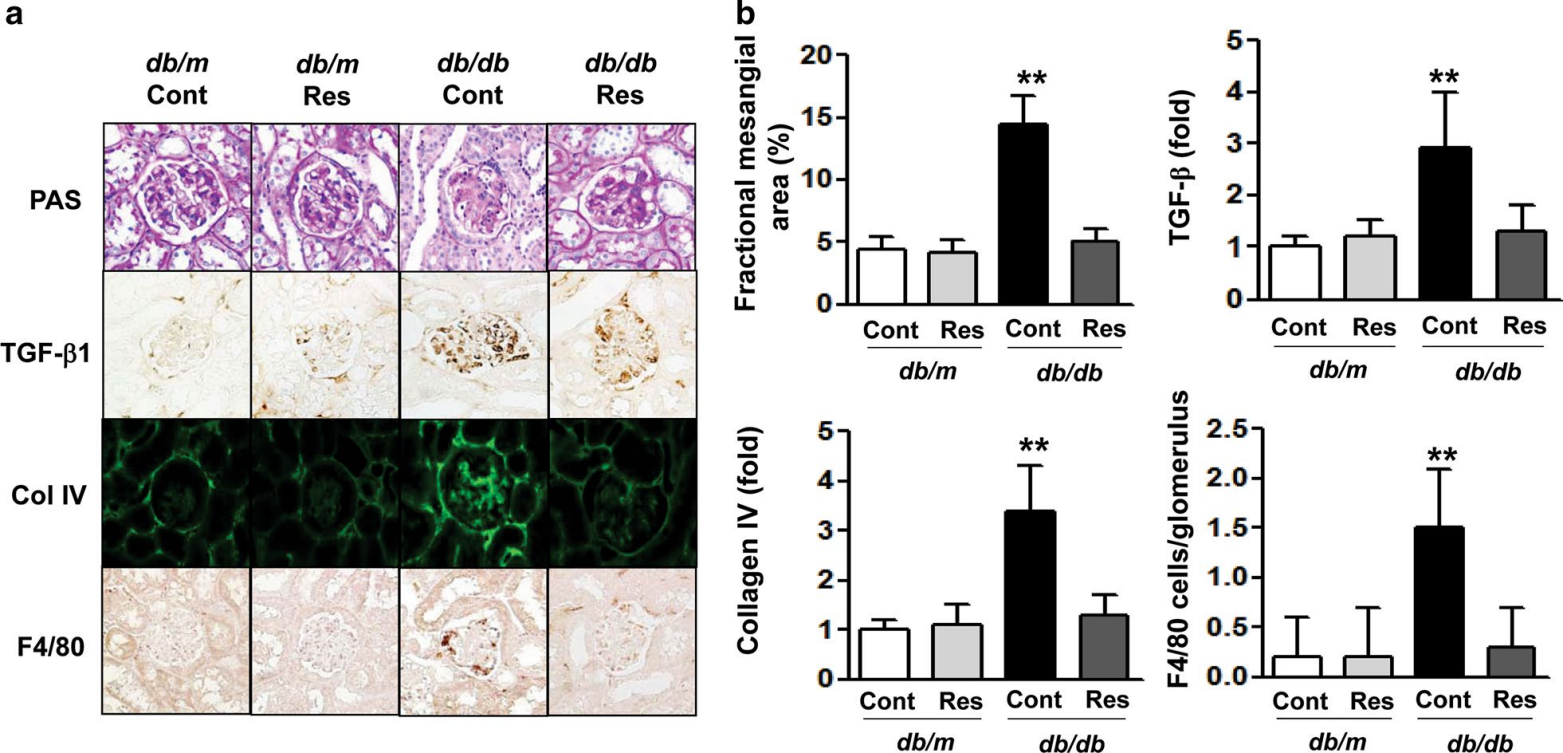
Changes in glomerular phenotypes in resveratrol-treated *db/db* mice. Glomerular mesangial fractional area, profibrotic TGF-β1, Col IV expression and F4/80-positive cell infiltration in the glomerulus of the cortical area of *db/m* and *db/db* mice with or without resveratrol treatment. **a** Representative sections stained with PAS reagent and immunohistochemical staining for TGF-β1, Col IV and F4/80-positive cells are shown (original magnification ×400). **b** Quantitative analyses of the results for the mesangial fractional area (%), TGF-β1, Col IV and F4/80-positive cells are shown. **p < 0.01 vs. *db/m*, *db/m* Res and *db/db* Res mice. *Res* resveratrol, *PAS* periodic acid–Schiff, *TGF-β1* transforming growth factor- β1, *Col IV* type IV collagen

There was a marked increase in the mesangial area in *db/db* mice compared with *db/m* mice (Fig. 1a, b, **p < 0.01). Consistent with the change in the mesangial fractional area, expression of TGF-β1, which is associated with extracellular matrix Col IV expression, and inflammatory cell infiltration in the glomerular area were significantly increased in *db/db* mice compared with *db/m* mice (Fig. 1a, b, **p < 0.01). All diabetes-induced renal phenotypic changes and inflammation shown in the *db/db* mice were ameliorated with resveratrol treatment. No changes were noted between the *db/m* and *db/m* Res mice.

### Renal cortical expression of AdipoR1 and AdipoR2, phospho-Thr^172^ and total AMPK, SIRT1, PPARα, and FoxOs

**Fig. 2:**
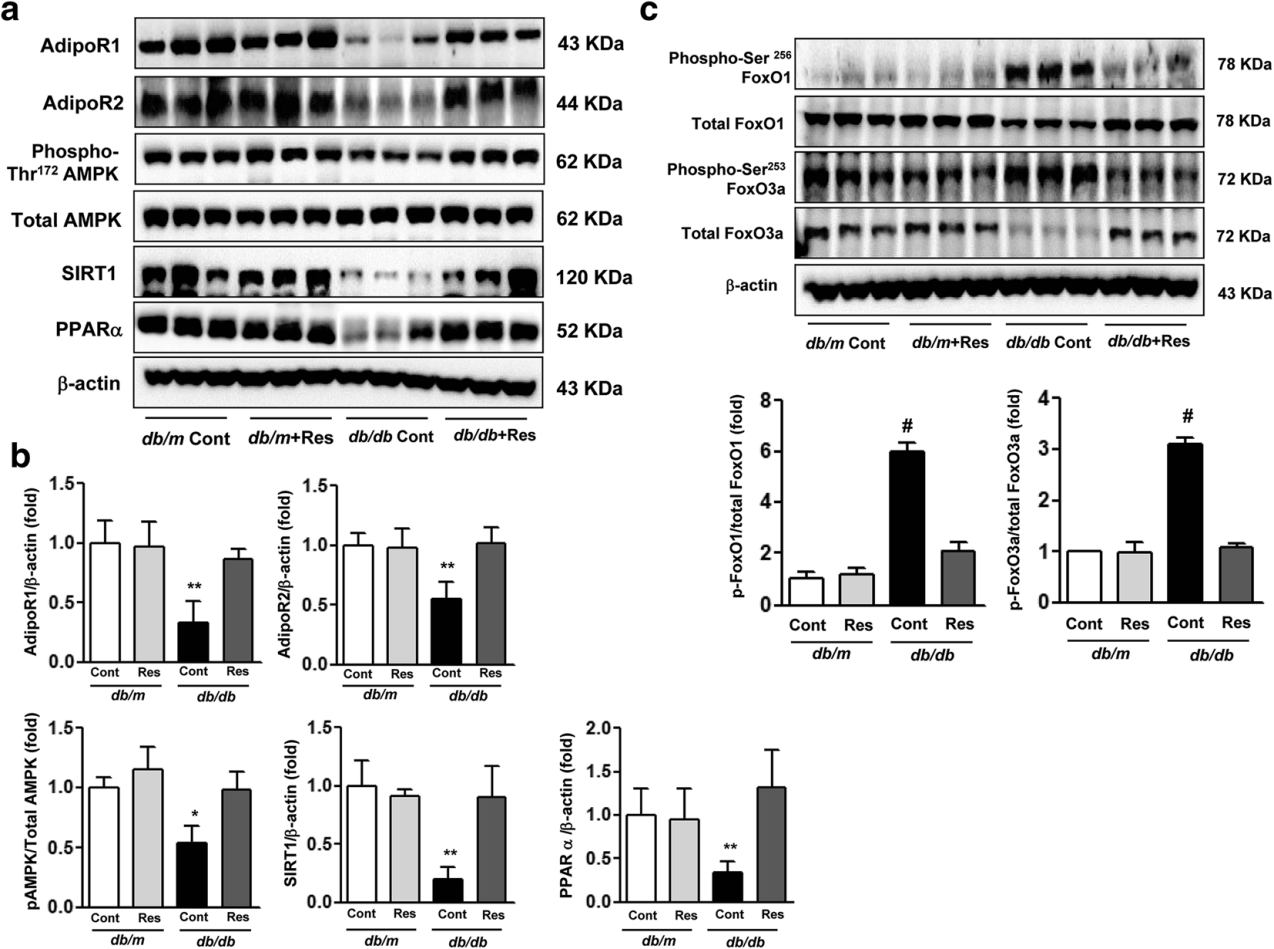
AdipoR1 and AdipoR2, phospho-Thr^172^ and total AMPK, SIRT1, PPARα and FoxO expression in the renal cortex of *db/m* and *db/db* mice with or without resveratrol. Protein lysates (40 μg) from renal cortex were separated by SDS–PAGE and analyzed by western blot. **a** Representative results are shown for AdipoR1 and AdipoR2, phospho-Thr^172^ and total AMPK, SIRT1, PPARα and β-actin. **b** Quantitative analyses of areas for AdipoR1 and AdipoR2, SIRT1 and PPARα, all relative to β-actin and phospho-Thr^172^ AMPK/total AMPK. *p < 0.05 and **p < 0.01 vs. db/m, db/m Res and db/db Res mice respectively. **c** Representative results for phospho-Ser^256^ and total FoxO1 and phospho-Ser^253^ and total FoxO3a and β-actin; quantitative analyses are for phosphorylated relative to total protein. ^#^p < 0.001 vs. *db/m*, *db/m* Res and *db/db* Res mice. *Adipo R* adiponectin receptor, AMPK 5′-adenosine monophosphate-activated protein kinase, *SIRT1* silent information regulator T1, *PPARα* peroxisome proliferator-activated receptorα, *Res* resveratrol, *FoxO* class O forkhead box

The expression of AdipoR1 and AdipoR2 levels in the renal cortex was markedly decreased in *db/db* mice compared with *db/m* and *db/m* Res mice, as assessed by western blot (Fig. 2a, b, **p < 0.01). Resveratrol treatment restored AdipoR1 and AdipoR2 levels in *db/db* mice to the levels in *db/m* and *db/m* Res mice. Phospho-Thr^172^ AMPK, a putative target of AdipoR1 and AdipoR2 and a principal downstream signal in the AdipoR1 pathway, and SIRT1 levels were significantly decreased in *db/db* mice compared with *db/m* mice (Fig. 2a, b). Interestingly, resveratrol treatment in *db/db* mice significantly increased the renal cortical expression of Phospho-Thr^172^ AMPK and SIRT1 (Fig. 2a, b, *p < 0.05 for AMPK and **p < 0.01 for SIRT1). Resveratrol did not affect the total AMPK expression in the renal cortex, suggesting that the alteration in AMPK phosphorylation was not the result of a reduction in total AMPK protein. The levels of PPARα, a principal downstream signal in the AdipoR2 pathway, were lower in the renal cortex of *db/db* mice compared with *db/m* and *db/m* Res mice, as assessed by western blot, and improved with resveratrol treatment (Fig. 2a, b, **p < 0.01). We examined whether AMPK phosphorylation and PPARα activation influenced downstream targets, specifically, phosphorylation of FoxOs in the kidney. Both phospho- Ser^256^ FoxO1 and Ser^253^ FoxO3a levels increased in *db/db* mice compared with *db/m* and db/m Res mice (Fig. 2c). Consistent with AMPK phosphorylation and PPARα activation, resveratrol treatment of *db/db* mice decreased the expression of both phosphorylated FoxO1 and FoxO3a in the renal cortex, resulting in increases of total FoxO1 and FoxO3a expression. This demonstrated that resveratrol positively regulates FoxO signaling in diabetic kidneys (Fig. 2c, ^#^p < 0.001).

### Altered renal expression of PGC-1α, ERR-1α, SREBP-1c, pACC, and intrarenal NEFA and TG

**Fig. 3:**
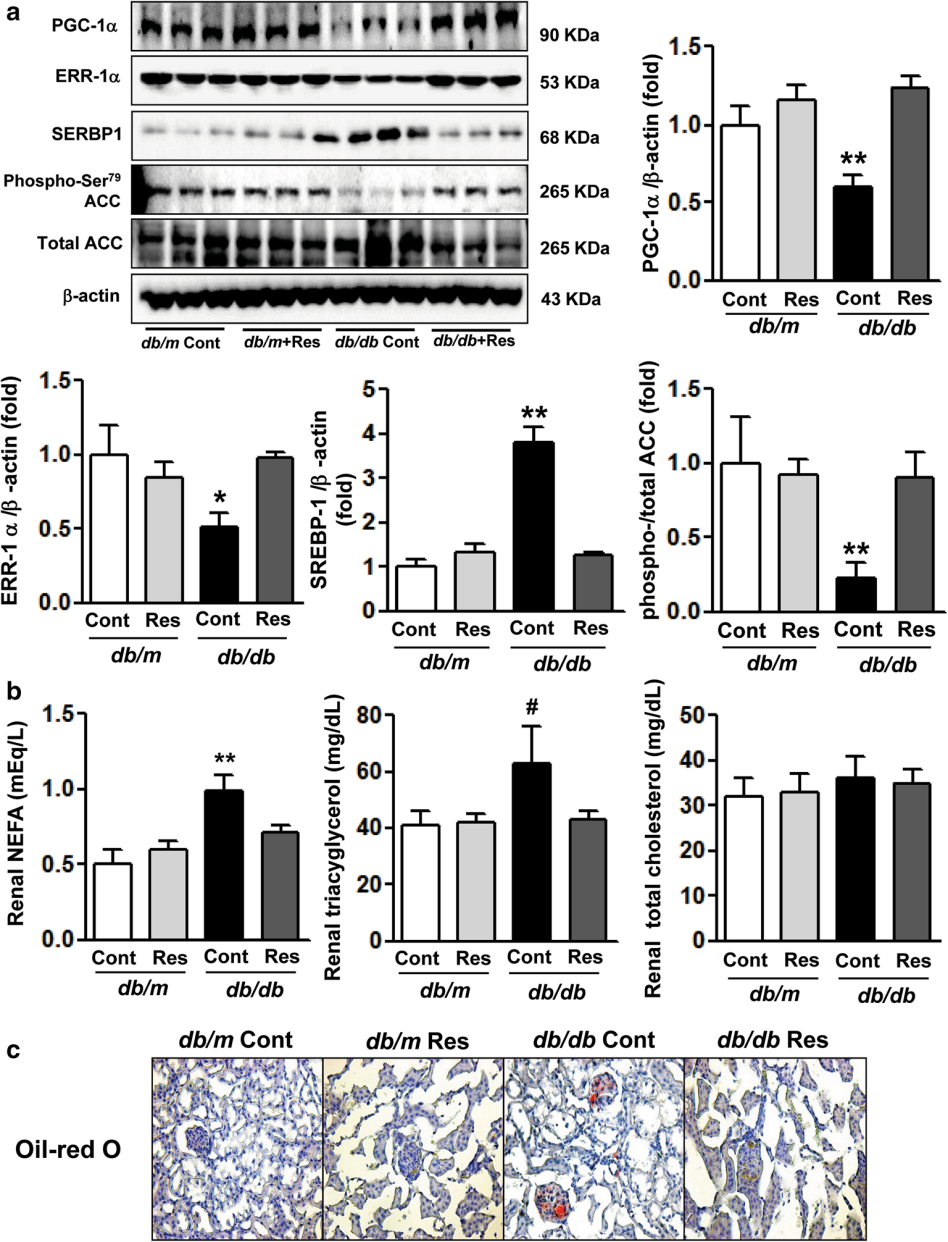
PGC-1α, ERR-1α, SREBP-1, pACC and intrarenal lipid levels in the renal cortex of the *db/m* and *db/db* mice with or without resveratrol. Protein lysates (40 μg) from renal cortexes were separated by SDS–PAGE and analyzed by western blot. **a** Representative results are for PGC-1α, ERR-1α, SREBP-1, pACC and β-actin and quantitative analyses of areas are for PGC-1α/β-actin, ERR-1α/β-actin, SREBP-1/β-actin, and pACC. *p < 0.05 and **p < 0.01 vs. *db/m*, *db/m* Res and *db/db* Res mice, respectively. **b** Quantitative analyses are for intrarenal NEFA, TG and total cholesterol concentrations. **p < 0.01 and ^#^p < 0.001 vs. *db/m*, *db/m* Res and *db/db* Res mice, respectively. **c** Oil red O staining from *db/m* and *db/db* mice with or without resverastrol (original magnification ×400). PGC-1α PPARγ co-activator 1α, *ERR-1α* estrogen-related receptor-1α, *pACC* phosphorylated acetyl-CoA carboxylase, *SREBP 1c* sterol regulatory element-binding protein 1c, *Res* resveratrol, *NEFA* nonesterified fatty acid, *TG* triacylglycerol

The PGC-1α–ERR-1α pathway and the downstream signaling SREBP-1c and pACC are primary targets of FoxOs and are critical for regulation of lipid metabolism and accumulation of lipids in the kidney (lipotoxicity). In *db/db* mice, PGC-1α and ERR-1α expression in renal cortex was significantly decreased compared with that in db/m and db/m Res mice. The SREBP-1c level in the renal cortex of *db/db* mice was increased compared with that in db/m and db/m Res mice, while the expression of pACC was reciprocally decreased (Fig. 3a, **p < 0.01). Inactivation of PGC-1α–ERR-1α and the subsequent increase in SREBP-1c and reciprocal decrease in pACC expression were associated with increases in the NEFA and TG content of the renal cortex of *db/db* mice (Fig. 3b, **p < 0.01 for NEFA and ^#^p < 0.001 for TG). Conversely, resveratrol treatment reactivated PGC-1α–ERR-1α signaling, which decreased SREBP-1c and increased pACC expression in the renal cortex of *db/db* mice, accompanied by decreased NEFA and TG accumulation in the tissues. These data suggested that resveratrol treatment prevented lipotoxicity in the kidneys of animal with type 2 diabetes. Oil red O staining of renal tissues showed no apparent lipid deposition in the kidneys of *db/m* control mice, regardless of whether they received resveratrol. Conversely, resveratrol treatment inhibited the accumulation of oil red O-stainable lipid in the glomerulus in *db/db* mice (Fig. 3c).

### Renal expression of eNOS, proapoptotic Bax, antiapoptotic Bcl-2 and TUNEL-positive cells

**Fig. 4:**
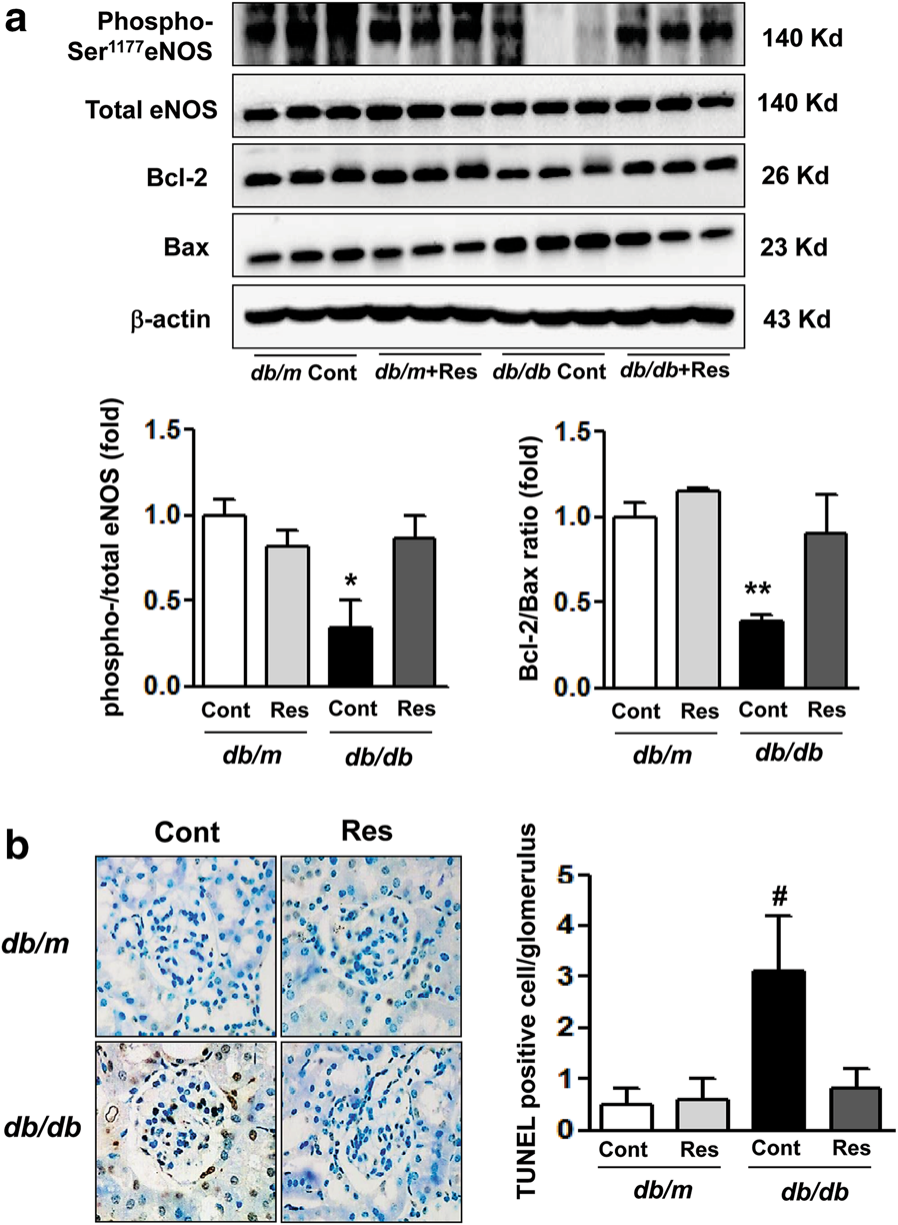
Expression of eNOS, proapoptotic Bax and antiapoptotic Bcl-2 in the renal cortex of *db/m* and *db/db* mice with or without resveratrol. Protein lysates (40 μg) from renal cortexes were separated by SDS–PAGE and analyzed by western blot. **a** Representative results are for phospho-Ser^1177^ and total-eNOS, Bax, Bcl-2 and β-actin and the quantitative analyses are for the phospho-Ser^1177^/total-eNOS ratio and Bcl-2/Bax ratio. *p < 0.05 and **p < 0.01 vs. *db/m*, *db/m* Res and *db/db* Res mice mice, respectively. **b** Representative immunohistochemical staining for TUNEL-positive cells with quantitative analysis. ^#^p < 0.001 vs. *db/m*, *db/m* Res and *db/db* Res mice. *eNOS* endothelial nitric oxide synthase, *Bcl-2* B cell leukemia/lymphoma 2, *Bax* Bcl-2-associated X protein, *Res* resveratrol, *TUNEL* terminal deoxynucleotidyl transferase dUTP nick end labeling

In *db/db* mice, consistent with inactivation of AMPK, PPARαα and FoxOs, the level of phospho-Ser^1177^ eNOS decreased. It was restored by resveratrol treatment, which thereby restored the phospho-Ser^1177^/total eNOS ratio (Fig. 4a, *p < 0.05). Also in *db/db* mice, Bax protein increased, whereas Bcl-2 protein decreased compared with *db/m* and *db/m* Res mice, resulting in an increase in TUNEL-positive cells in the kidneys (Fig. 4a, b, **p < 0.01 and ^#^p < 0.001). Resveratrol treatment of *db/db* mice decreased Bax expression and reciprocally increased that of Bcl-2, leading to the restoration of the Bcl-2/Bax ratio, which was associated with a decrease in TUNEL-positive cells in the kidneys. These changes were consistent with the increased phosphorylation of eNOS in *db/db* and *db/db* Res mice, which attenuated apoptotic cell death in renal cells of diabetic animals.

### Effects of resveratrol on renal and 24-h urinary 8-OH-dG and isoprostane

**Fig. 5:**
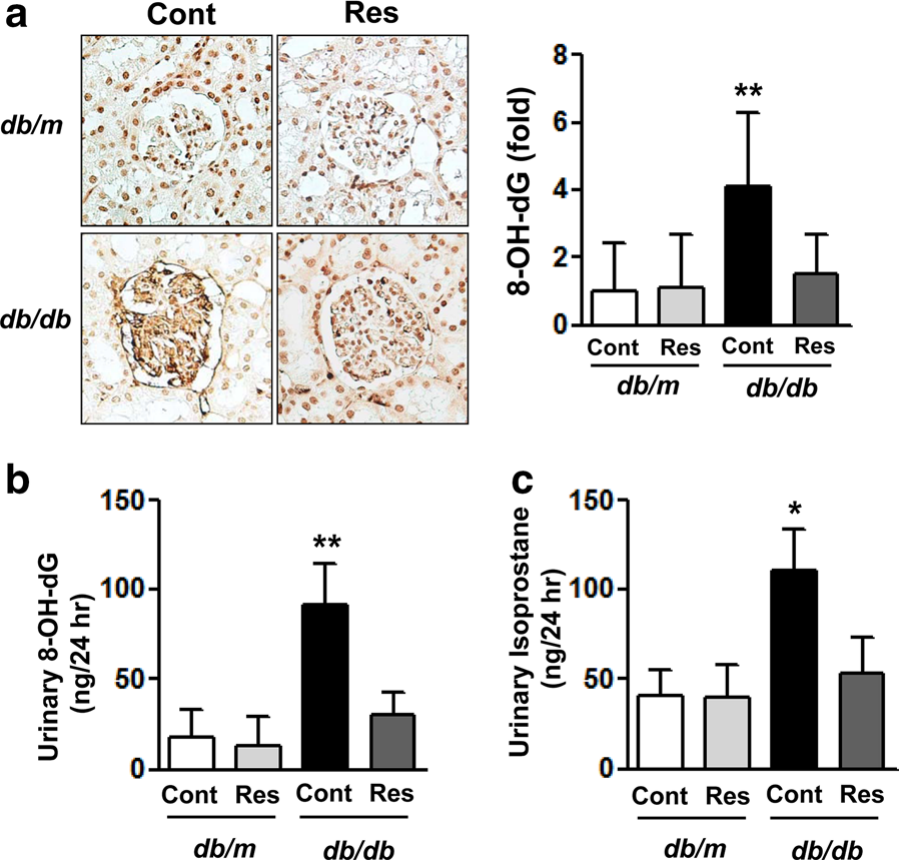
Intrarenal and 24-h urinary 8-OH-dG and isoprostane levels in *db/m* and *db/db* mice with or without resveratrol. **a** Representative immunohistochemical staining for 8-OH-dG and quantitative analysis. **b** **p < 0.01 vs. *db/m*, *db/m* Res and *db/db* Res mice. Levels of 24-h urinary 8-OH-dG (**c**) and isoprostane (**d**). *p < 0.05 and **p < 0.01 vs. *db/m*, *db/m* Res and *db/db* Res mice, respectively. *8-OH-dG* 8-hydroxy-2′-deoxyguanosine, *Res* resveratrol

We evaluated the oxidative stress and lipid peroxidation caused by renal damage in type 2 diabetes using renal and 24-h urinary 8-OH-dG and 24-h urinary 8-isoprostane levels. In *db/db* mice, renal and 24-h urinary 8-OH-dG and 24-h urinary 8-isoprostane levels were significantly elevated compared with those of *db/m* mice (Fig. 5a–c, *p < 0.05 and **p < 0.01). Resveratrol treatment of *db/db* mice significantly diminished both renal and urinary 8-OH-dG and urinary 8-isoprostane. These findings suggested that the exacerbated renal oxidative stress and lipid peroxidation in type 2 diabetic *db/db* mice could be ameliorated by resveratrol.

### In vitro studies

**Fig. 6:**
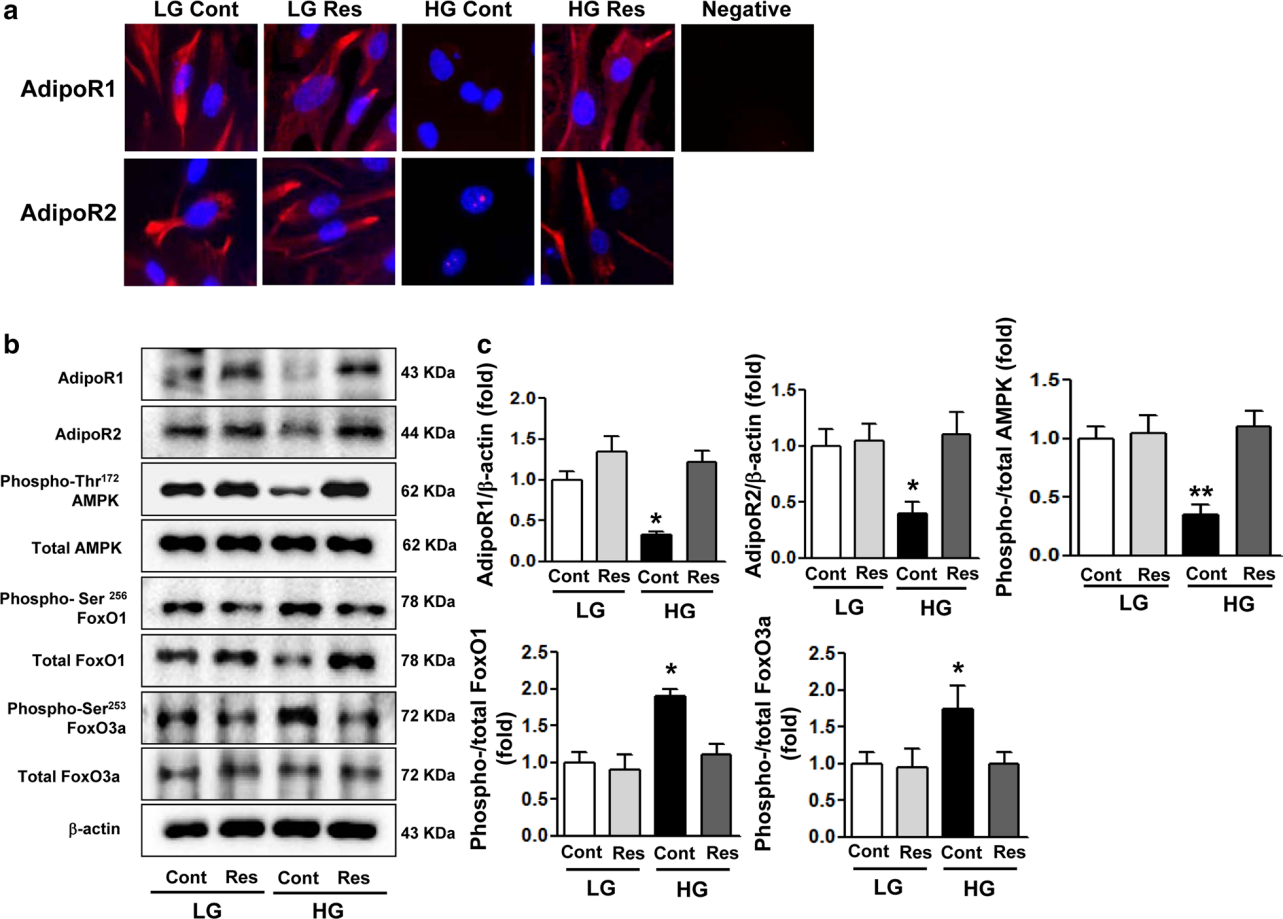
**a** Immunofluorescence analysis was performed for AdipoR1 and AdipoR2 in the HGECs with or without resveratrol treatment (original magnification, ×400). Effect of resveratrol on intracellular signaling in HGECs cultured in low glucose (LG, 5 mmol/L of d-glucose) or high glucose (HG, 30 mmol/L of d-glucose) with or without resveratrol (50 μM). AdipoR1 and AdipoR2, phospho-Thr^172^ AMPK, total AMPK, phospho-Ser^256^ FoxO1, total FoxO1, phospho-Ser^253^ FoxO3a and total FoxO3a were assessed in HGECs. Protein lysates (10 μg) were separated by SDS–PAGE and analyzed by western blot. **b** Representative results for AdipoR1 and AdipoR2, phospho-Thr^172^ AMPK, total AMPK, phospho-Ser^256^ FoxO1 and total FoxO1, phospho-Ser^253^ FoxO3 and total FoxO3a and β-actin. **c** Quantitative analyses for AdipoR1/β-actin, AdipoR2/β-actin and phospho-Thr^172^ AMPK, phospho-Ser^256^ FoxO1 and phospho-Ser^253^ FoxO3a, all of which are relative to totals. *p < 0.05 and **p < 0.01 compared with LG, LG + Res, and HG + Res. *HGECs* human glomerular endothelial cells, *AdipoR* adiponectin receptor, *AMPK* 5′-adenosine monophosphate-activated protein kinase, *FoxO* class O forkhead box, *LG* low glucose, *Res* resveratrol, *HG* high glucose

Next, we examined whether resveratrol treatment of HGECs could upregulate the expression of AdipoR1 and AdipoR2 under conditions of high-glucose-induced oxidative stress. Immunofluorescence analysis showed that the expression of AdipoR1 and AdipoR2 was lower in high-glucose (30 mmol/L d-glucose) medium than in low-glucose (5 mmol/L d-glucose) medium. Interestingly, resveratrol treatment significantly increased the AdipoR1 and AdipoR2 expression of HGECs in high-glucose conditions, but not in low-glucose conditions (Fig. 6a). We also examined whether resveratrol-mediated AdipoR1 and AdipoR2 activation of HGECs resulted in activation of AMPK, PPARα and FoxOs. HGECs exposed to high-glucose medium significantly decreased the expression of AdiopoR1 and AdipoR2 and their downstream signaling, i.e., phospho-Thr^172^ AMPK, total FoxO1 and FoxO3a (Fig. 6b, c). Consistent with the in vivo studies, resveratrol reversed the high-glucose-induced changes to the values seen with low-glucose medium.

**Fig. 7:**
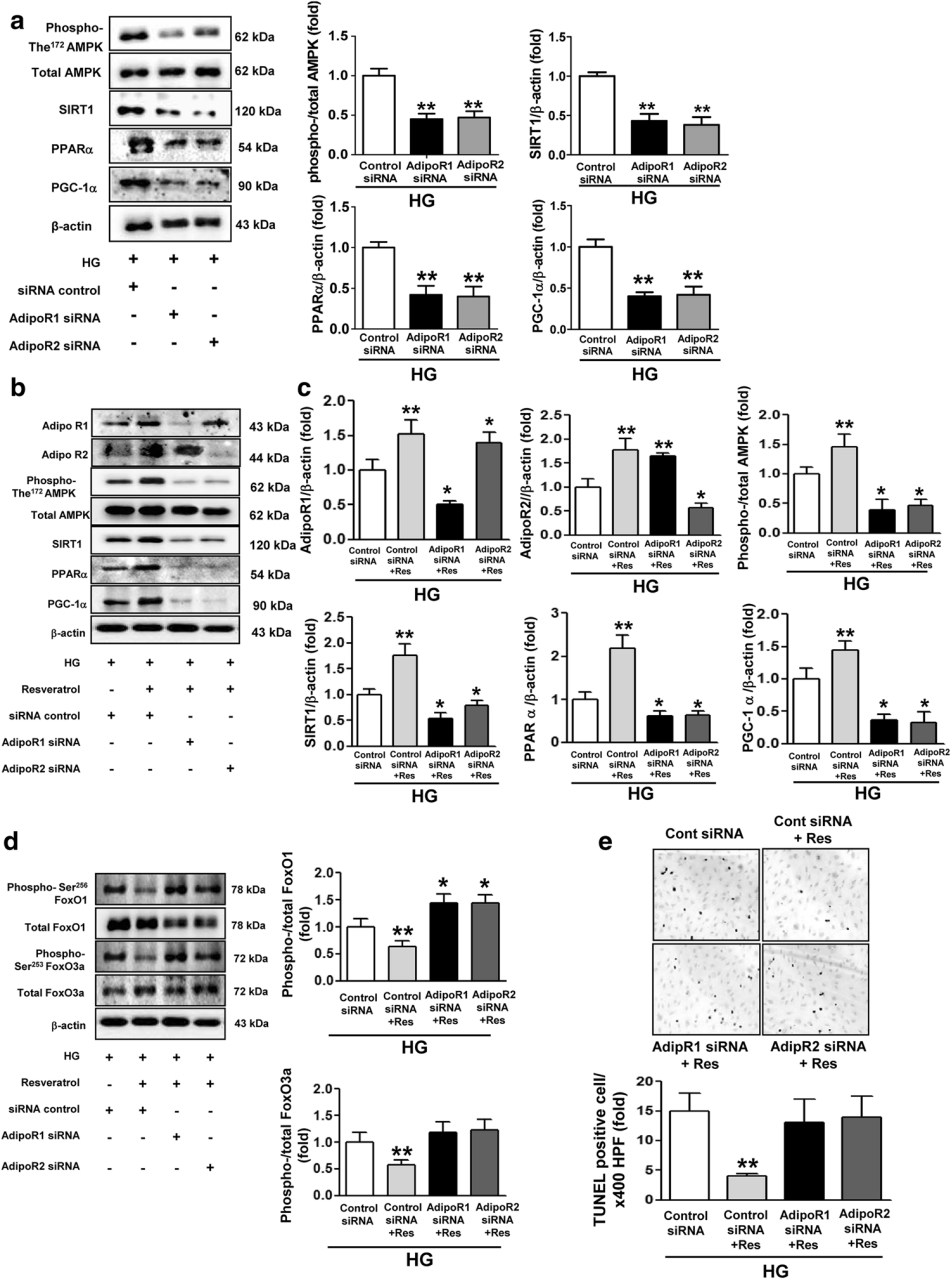
The effect of resveratrol on the activities of AMPK–SIRT1–PGC-1α signaling, PPARα, FoxO, and apoptosis in HGECs exposed to high-glucose medium. Cultured HGECs in HG with or without resveratrol were transfected with 50 nmol/L control siRNA, 50 nmol/L AdipoR1 or AdipoR2 siRNA using transfection reagent (G-Fectin). Approximately 48 h after transfection, protein lysates (10 μg) were analyzed by Western blot for AdipoR1 and AdipoR2, phospho-Thr^172^ AMPK, total AMPK, SIRT1, PGC-1α, and PPARα. **a** Representative results for phospho-Thr^172^ AMPK, total AMPK, SIRT1, PPARα, PGC-1α and β-actin and quantitative analyses for SIRT1, PPARα and PGC-1α relative to β-actin and phospho-Thr^172^ AMPK/total AMPK in HGECs exposed to high-glucose medium without resveratrol. **p < 0.01 compared with HG + siRNA control. **b** Representative results for AdipoR1 and AdipoR2, phospho-Thr^172^ AMPK, total AMPK, SIRT1, PPARα, PGC-1α and β-actin. **c** Quantitative analyses for AdipoR1 and AdipoR2, SIRT1, PPARα and PGC-1α relative to β-actin and phospho-Thr^172^ AMPK/total AMPK **d** Representative results for phospho-Ser^256^ FoxO1 and total FoxO1, phospho-Ser^253^ FoxO3a and total FoxO3a and β-actin and quantitative analyses for phospho-Ser^256^ FoxO1/total FoxO1 and phospho-Ser^253^ FoxO3a/total FoxO3a. *p < 0.05 compared with HG + siRNA control. **p < 0.05 compared with HG + siRNA control and p < 0.01 compared with the other groups. **e** Representative immunohistochemical staining for TUNEL-positive HGECs with quantitative analysis. **p < 0.01 compared with the other groups. *AdipoR* adiponectin receptor, *AMPK* 5′-adenosine monophosphate-activated protein kinase, *SIRT1* silent information regulator T1, *PPARα* peroxisome proliferator-activated receptorα, *PGC-1α* PPARγ co-activator 1α, *HG* high glucose, *Res* resveratrol, *siRNA* small interfering RNA, *FoxO* class O forkhead box

Previous studies showed that AdipoR1 activates AMPK phosphorylation and AdipoR2 activates PPARα in the liver and muscle [17, 18]. Therefore, we evaluated the role of adiponectin receptors in mediating the activation of the downstream molecules, AMPK, SIRT1, PPARα, PGC-1α, and FoxOs, in resveratrol-treated HGECs that were cultured in high-glucose medium. For this, we performed additional experiments using siRNAs against *AdipoR1* and *AdipoR2*. The siRNAs could efficiently block the expression of both AdipoR1 and AdipoR2 (Additional file 2: Figure S1). The expressions of phosphorylated AMPK, SIRT1, PGC-1α, and PPARα in high-glucose medium-exposed HGECs were significantly attenuated by siRNA-mediated knockdown of AdipoR1 or AdipoR2 in the cells (Fig. 7a). Next, we investigated the resveratrol-induced changes of AMPK, SIRT1, PGC-1α, and PPARα in high-glucose medium-exposed HGECs and underlying mechanisms. The results showed that resveratrol treatment in high-glucose medium-exposed HGECs can increase the activities of AMPK, SIRT1, PGC-1α, and PPARα in those cells and these effects were dependent on AdipoR1 and AdipoR2 inducing properties of resveratrol (Fig. 7b, c). Furthermore, resveratrol treatment in HGECs exposed to high-glucose medium significantly increased the dephosphorylation of FoxO1 and FoxO3a, when compared with untreated cells (Fig. 7d). This is consistent with the in vivo results shown in Fig. 2c. Also, the FoxO signaling activation by resveratrol treatment was dependent on its AdipoR1 and AdipoR2 inducing effects (Fig. 7d). In vivo studies (as shown in Fig. 4b) demonstrated the antiapoptotic effects of resveratrol in renal cells of type 2 diabetic *db/db* mice. Therefore, we investigated whether the antiapoptotic effect of resveratrol under high-glucose conditions was AdipoR1 or AdipoR2 dependent. The results showed that a decrease in TUNEL-positive cells in HGECs with resveratrol was significantly reversed by AdipoR1 and AdipoR2 siRNA treatment (Fig. 7e). These results suggested that resveratrol may increase the expression of both AdipoR1 and AdipoR2 in the kidney and consequently result in the activation of AMPK and PPARα and their downstream signals including PGC-1α and FoxOs.

## Discussion

Deletion of the gene for adiponectin accelerates DN in Akita mice [19]. However, adiponectin reduces microalbuminuria and provides renoprotective effects by improving endothelial dysfunction and uncoupling the vascular endothelial growth factor–nitric oxide axis in rats with streptozotocin-induced type 1 diabetes [20]. Adiponectin also retards the progression of DN in *db/db* mice by counteracting angiotensin II [21]. However, few therapeutic agents activate adiponectin, especially in the kidney [14, 22]. Therefore, we investigated resveratrol as a promising agent for preventing and treating DN through increasing AdipoR1 and AdipoR2 expression.

Resveratrol is an activator of AMPK–SIRT1–PPARα, a key to the regulation of lipids and glucose homeostasis and control of oxidative stress. Adiponectin is a 30-kDa circulating plasma protein primarily secreted by adipocytes. Low circulatory levels seem to contribute to the pathophysiology of insulin resistance, type 2 DM and cardiovascular disease in obese or overweight patients or people with type 2 DM [8]. Resveratrol has beneficial effects against development of albuminuria in animal experiments [5–7]. Ji et al. reported that resveratrol increases expression of AdipoR1 by activating FoxO1 in rats with streptozotocin-induced diabetes and mesangial cell cultures [14]. Another report found that decreased plasma adiponectin levels and renal expression of AdipoR1 but not AdipoR2 protein are associated with oxidative stress in rats with streptozotocin-induced diabetes [23]. However, the effects of resveratrol on adiponectin receptors in the kidneys of individuals with type 2 DM are not well known.

Previously, we showed that resveratrol prevents DN via activation of the AMPK–SIRT1–PGC-1α axis and that PPARα was activated by resveratrol in *db/db* mice [24]. These findings prompted us to investigate whether the renoprotective effect of resveratrol in DN was associated with adiponectin receptor activation, because AdipoR1 activates the AMPK pathway and AdipoR2 activates the PPARα pathway. AdipoR1 is expressed predominantly in skeletal muscle and mediates fatty acid oxidation and glucose uptake by activating AMPK–SIRT1–PGC-1α and Ca^2+^ signaling pathways, which can be also activated by exercise. Both AdipoR1 and AdipoR2 are expressed in the liver and regulate glucose and lipid metabolism by activating the AMPK and PPARα pathways [17]. Sharma et al. found that in the kidney, AdipoR1 is expressed to a similar degree as in liver, but AdipoR2 showed lower renal expression in in vitro experiments using mouse podocytes [6]. This group also found that adiponectin administration activated AMPK and decreased albuminuria by reducing oxidative stress. Guo and Zhao showed that AdipoR1 expression was significantly decreased in rats with streptozotocin-induced diabetes compared with controls, but there were no significant differences in AdipoR2 expression between the two groups, although expression in the diabetic group seemed lower than in controls [23]. These findings suggested that adiponectin exerts its effect in kidneys via AdipoR1 rather than AdipoR2. Okada-Iwabu et al. demonstrated the efficacy of AdipoRon, an oral active AdipoR agonist, and showed that AdipoR1- and AdipoR2-knockout *db/db* mice had a shorter survival than wild-type, AdipoR1 knockout or AdipoR2 knockout *db/db* mice [22]. In their study, AdipoR1-knockout *db/db* mice had shorter survival than AdipoR2-knockout mice and AdipoR2-knockout *db/db* mice had shorter survival than wild-type *db/db* mice. Yu et al. showed that AdipoR1 and AdipoR2 expression in rats with CKD were significantly increased, as were serum and urine adiponectin levels [25]. Therefore, AdipoR1 and AdipoR2 activation appears to contribute to protection against CKD progression including in DN, although AdipoR1 is more highly expressed than AdipoR2 in kidneys and the relationship between renal expression of renal AdipoR1 and AdipoR2 and renal function is biphasic. A negative relationship exists in the early stages of DN such as microalbuminuria, and a positive association in more advanced renal damage, indicating that the counteracting upregulation of renal AdipoR1 and AdipoR2 mitigates renal damage [26].

In this study, we found that DN in type 2 DM was characterized by suppression of AdipoR1, AdipoR2 and AMPK–SIRT1–PPARα expression, and inactivation of FoxO1 and FoxO3a. Together, these effects led to lipotoxicity, endothelial dysfunction, oxidative stress and increased apoptosis in the kidney. Resveratrol treatment reversed these changes, improving function and phenotypes such as albuminuria and mesangial expansion and inflammation. In addition, in HGECs, which express both AdipoR1 and AdipoR2, resveratrol treatment reversed the high-glucose-induced decreases in the expression of AdipoR1 and AdipoR2 and their downstream signals, such as AMPK–SIRT1, PPARα, PGC-1α, and FoxOs to the levels observed in low-glucose medium. There is controversy whether resveratrol first induces AdipoR1 and AdipoR2, which then activate the AMPK–SIRT1–PGC1α pathway, or whether resveratrol activates FoxOs, which are responsible for AdipoR1 and AdipoR2 expression. In the current study, we used AdipoR1 siRNA and AdipoR2 siRNA to clarify the effect of resveratrol on the expression of AMPK and FoxOs. The results showed that resveratrol-induced expression of AMPK and of FoxO1 and FoxO3a expressions was decreased by AdipoR1 siRNA and AdipoR2 siRNA, respectively. Therefore, we suggest that the protective role of resveratrol seems to be mediated through direct activation of both AdipoR1 and AdipoR2, which in turn increases the expression of AMPK and FoxOs.

In our study, AdipoR1 and AdipoR2 expression decreased and AMPK and PPARα were inactivated in *db/db* mice. Conversely, resveratrol administration restored AdipoR1 and AdipoR2 expression and activated AMPK–SIRT1–PGC-1α, which decreased phosphorylation of FoxO1 and FoxO3a [27]. In that study, Tsuchida et al. demonstrated that AdipoR1 and AdipoR2 expression is controlled via the FoxO1 pathway in skeletal muscle and adipose tissue in mice with streptozocin-induced diabetes. FoxOs, downstream effectors shared by both AMPK–SIRT1–PGC-1α signaling and PPARα activation, may explain this interaction [28, 29]. We and others demonstrated that diabetic conditions, which are usually associated with increased TGF-β levels, induce PI3 K and Akt activation, leading to FoxO3a phosphorylation and its nuclear exclusion and inactivation in both streptozotocin-induced type 1 diabetes and type 2 diabetic *db/db* mice [2, 30]. In our study, activation of PGC-1α–ERR-1α–pACC and suppression of SREBP-1c decreased lipotoxicity, oxidative stress and apoptosis in kidneys. Studies of DN in human and animals showed that accumulation of lipids in the kidney and lipotoxicity might be related to oxidative stress and apoptosis and possibly involved in diabetic CKD pathogenesis-related FoxO inactivation [2, 31, 32].

Decreased plasma adiponectin levels are associated with obesity, insulin resistance and type 2 DM. Replenishment of adiponectin decreases insulin resistance, glucose intolerance and tissue triglyceride content by increasing fatty acid oxidation and decreasing oxidative stress and inflammation [22]. Adiponectin is critical in the development of alcoholic fatty liver disease. In a study by Ajmo et al., resveratrol protected against alcoholic fatty liver disease in mice by activating AMPK–SIRT1–PGC-1α and increasing hepatic AdipoR1/R2 expression [13]. Therefore, adiponectin and its receptors including AdipoR1 and AdipoR2 might be involved in lipid metabolism in the kidney as well as in adipose tissue and the liver. However, a number of serious concerns have been raised about adiponectin as a therapeutic agent for diabetes and its complications. Overexpression of adiponectin in genetically engineered mice and stimulators of adiponectin secretion such as thiazolidinediones [33] and fibroblast growth factor 21 [34] are associated with reduced bone density, heart damage (left ventricular hypertrophy), weight gain from promoting adipogenesis and angiogenesis [35], and infertility [36]. Therefore, resveratrol may be a good candidate for increasing adiponectin levels and activating adiponectin actions in patients with type 2 diabetic CKD without potential side effects. Another interesting finding in this study was the lack of effect of resveratrol on AdipoR1 and AdipoR2 expression in *db/m* mice or in low-glucose conditions, but its ability to restore AdipoR1 and AdipoR2 expression in *db/db* mice or in high-glucose condition. It has been reported that resveratrol usually has protective roles in DN only when adiponectin receptor expression is decreased [14]. Although its exact mechanisms of action are still controversial, resveratrol-induced AMPK activation appears to be dose, time, and stress dependent [37, 38].

## Conclusions

The results of this study indicated that resveratrol increased circulating adiponectin levels and protected against DN by ameliorating inflammation, oxidative stress, apoptosis and endothelial dysfunction via activating AMPK–SIRT1–PPARα through AdipoR1 and AdipoR2. The results supported in vitro data in HGECs that upregulation of AdipoR1 and R2 by resveratrol in cells grown in high-glucose media could be a key response that protects against glucolipotoxicity in the kidney. These results suggest that resveratrol may be a promising therapeutic agent for type 2 DN.

## Additional files

10.1186/s12967-016-0922-9 Detailed information for western blot analysis and method using cultured human glomerular endothelial cells in in vitro study.
10.1186/s12967-016-0922-9 Downregulation of AdipoR1 and AdipoR2 expression in HGECs by siRNA. Levels of AdipoR1 and AdipoR2 expression were measured by Western blot. **p < 0.01 compared with control (scrambled) siRNA.

## Abbreviations

AMPK: 5′-adenosine monophosphate-activated protein kinase
AdipoR: adiponectin receptor
Bax: Bcl-2-associated X protein
Bcl-2: B cell leukemia/lymphoma 2
CKD: chronic kidney disease
CMC: carboxymethyl cellulose sodium salt
Col IV: type IV collagen
DAPI: 4,6-diamidino-2-phenylindole
DM: diabetes mellitus
DN: diabetic nephropathy
eNOS: endothelial nitric oxide synthase
ERR: estrogen-related receptor
8-epi-PGF2: 8-epi-prostaglandin F2
8-OH-dG: 8-hydroxy-2-deoxyguanosine
FoxO: class O forkhead box
HbA1c: hemoglobin A1c
HGEC: human glomerular endothelial cell
NEFA: nonesterified fatty acid
pACC: phosphorylated acetyl-CoA carboxylase
PAS: periodic acid–Schiff
PBS: phosphate buffered saline
PGC: PPARγ coactivator
PPAR: peroxisome proliferator-activated receptor
siRNA: small interfering RNA
SIRT1: silent information regulator T1
SREBP: sterol regulatory element-binding protein
TG: triacylglycerol
TGF-β1: transforming growth factor-β1
TUNEL: terminal deoxynucleotidyl transferase-mediated dUTP nick-end labeling
