# Erratum to: Resveratrol increases AdipoR1 and AdipoR2 expression in type 2 diabetic nephropathy

**DOI:** 10.1186/s12967-016-0967-9

**Published:** 2016-07-13

**Authors:** Hoon Suk Park, Ji Hee Lim, Min Young Kim, Yaeni Kim, You Ah Hong, Sun Ryoung Choi, Sungjin Chung, Hyung Wook Kim, Bum Soon Choi, Yong Soo Kim, Yoon Sik Chang, Cheol Whee Park

**Affiliations:** Division of Nephrology, Department of Internal Medicine, College of Medicine, The Catholic University of Korea, Seoul, Republic of Korea; Department of Internal Medicine, Seoul St. Mary’s Hospital, The Catholic University of Korea, #505, Banpo-Dong, Seocho-Ku, Seoul, 137-040 Republic of Korea

## Erratum to: J Transl Med (2016) 14:176 DOI 10.1186/s12967-016-0922-9

Unfortunately, the original version of this article [1] contained an error. The funding reference was incorrect. The correct funding information can be found in this erratum.
