# Supplementary material for: Resveratrol increases AdipoR1 and AdipoR2 expression in type 2 diabetic nephropathy
Source: J Transl Med. 2016 Jun 11;14:176. doi: 10.1186/s12967-016-0922-9 (PMC4902973; doi:10.1186/s12967-016-0922-9)
Supplement: Supplementary file 1 — 10.1186/s12967-016-0922-9 Detailed information for western blot analysis and method using cultured human glomerular endothelial cells in in vitro study. [file 12967_2016_922_MOESM1_ESM.docx]

***Western blot analysis***

Total proteins of renal cortical tissues were extracted with Pro-Prep Protein Extraction Solution (Intron Biotechnology, Gyeonggi-Do, Korea), following the manufacturer’s instructions. Equal amounts of the samples were separated on SDS-PAGE and transferred to Nitrocellulose (NC) membranes (Millipore, Bedford, MA, USA). The membranes were blocked by 3% dry skim milk for 30 min and then incubated with antibodies for AdipoR1 (1:2000; Abcam, Cambridge, UK), AdipoR2 (1:2000; Abcam), phospho-Thr^172^ AMPK (1:2000; Cell Signaling Technology, Danvers, MA), total AMPK (1:3000; Cell Signaling Technology), silent information regulator T1(SIRT1) (1:2000; Cell Signaling Technology), PPARα (1:2000; Abcam), phospho-Ser^256^ class O forkhead box (FoxO)1 (1:2000; Cell Signaling Technology), total FoxO1 (1:2000; Cell Signaling Technology), phospho-Ser^253^ FoxO3a (1:2000; Abcam), total FoxO3a (1:2000; Abcam), PPARγ coactivator (PGC)-1α (1:2000; Novus Biologicals, Littleton, CO, USA), estrogen-related receptor (ERR)-1α (1:3000; Chemicon-Millipore, Billerica, MA, USA), sterol regulatory element-binding protein (SREBP)-1c (1:200; Santa Cruz Biotechnology, Santa Cruz, CA, USA), phosphorylated acetyl-CoA carboxylase (pACC) (1:300; Santa Cruz Biotechnology), total ACC (1:300; Santa Cruz Biotechnology), phospho-Ser^1177^ endothelial nitric oxide synthase (eNOS) (1:2000; Cell Signaling Technology), total eNOS (1:3000; Cell Signaling Technology), B cell leukemia/lymphoma 2 (Bcl-2) (1:200; Santa Cruz Biotechnology), Bcl-2-associated X protein (Bax) (1:300; Santa Cruz Biotechnology), and β-actin (1:10,000; Sigma-Aldrich, St Louis, MO, USA). After incubation with horseradish peroxidase-conjugated anti-mouse or anti-rabbit IgG (1:2000; Cell Signaling Technology), target proteins were visualized using an enhanced chemiluminescence substrate (ECL Plus; GE Healthcare Bio-Science, Piscataway, NJ, USA).

***Human glomerular endothelial cell (HGEC) culture and small interfering RNA (siRNA) transfection***

HGECs (Angio-Proteomie, Boston, MA, USA) were cultured in Endogrowth medium (Angio-Proteomie) at 37 °C in a humidified, 5% CO_2_/95% air atmosphere. Passages 4–8 were used in all experiments. HGECs were exposed to low glucose (5 mmol/L D-glucose) or high glucose (30 mmol/L D-glucose), with or without an additional 6-h application of resveratrol (50 μM). Western blots were performed using specific antibodies against AdipoR1 (Abcam), AdipoR2 (Abcam), phospho-Thr^172^ AMPK (Cell Signaling Technology), total AMPK (Cell Signaling Technology), phospho-Ser^256^ FoxO1 (Cell Signaling Technology), total FoxO1 (Cell Signaling Technology), phospho-Ser^253^ FoxO3a (Abcam), total FoxO3a (Abcam) and β-actin (Sigma-Aldrich). Small interfering RNAs (siRNAs) targeted to AdipoR1and AdipoR2 and scrambled siRNA (siRNA cont) were complexed with transfection reagent (Lipofectamine 2000; Invitrogen, Carlsbad, CA), according to the manufacturer’s instructions. The sequences of the siRNAs were: AdipoR1, 5′-GGACAACGACUAUCUGCUACATT-3′, AdipoR2, 5′-CCAACUGGAUGGUACACGA-3′, and nonspecific scrambled siRNA, 5′-CCUACGCCACCAAUUUCGU-3′ (Bioneer, Daejeon, Korea). HGECs in 6-well plates were transfected with a final concentration of 50 nM AdipoR1 and AdipoR2 siRNAs using Lipofectamine 2000 (Invitrogen) in OptiMEM(R) I reduced-serum medium (Gibco Invitrogen, Carlsbad, CA, USA) for 24 h. The medium was changed to growth medium for subsequent incubations. After transfection, cells were treated with resveratrol (50 μM) in high-glucose media to evaluate the effects of siRNAs on the reactions of HGECs.
