# Supplementary figures and images for: Resveratrol increases AdipoR1 and AdipoR2 expression in type 2 diabetic nephropathy
Source: J Transl Med. 2016 Jun 11;14:176. doi: 10.1186/s12967-016-0922-9 (PMC4902973; doi:10.1186/s12967-016-0922-9)

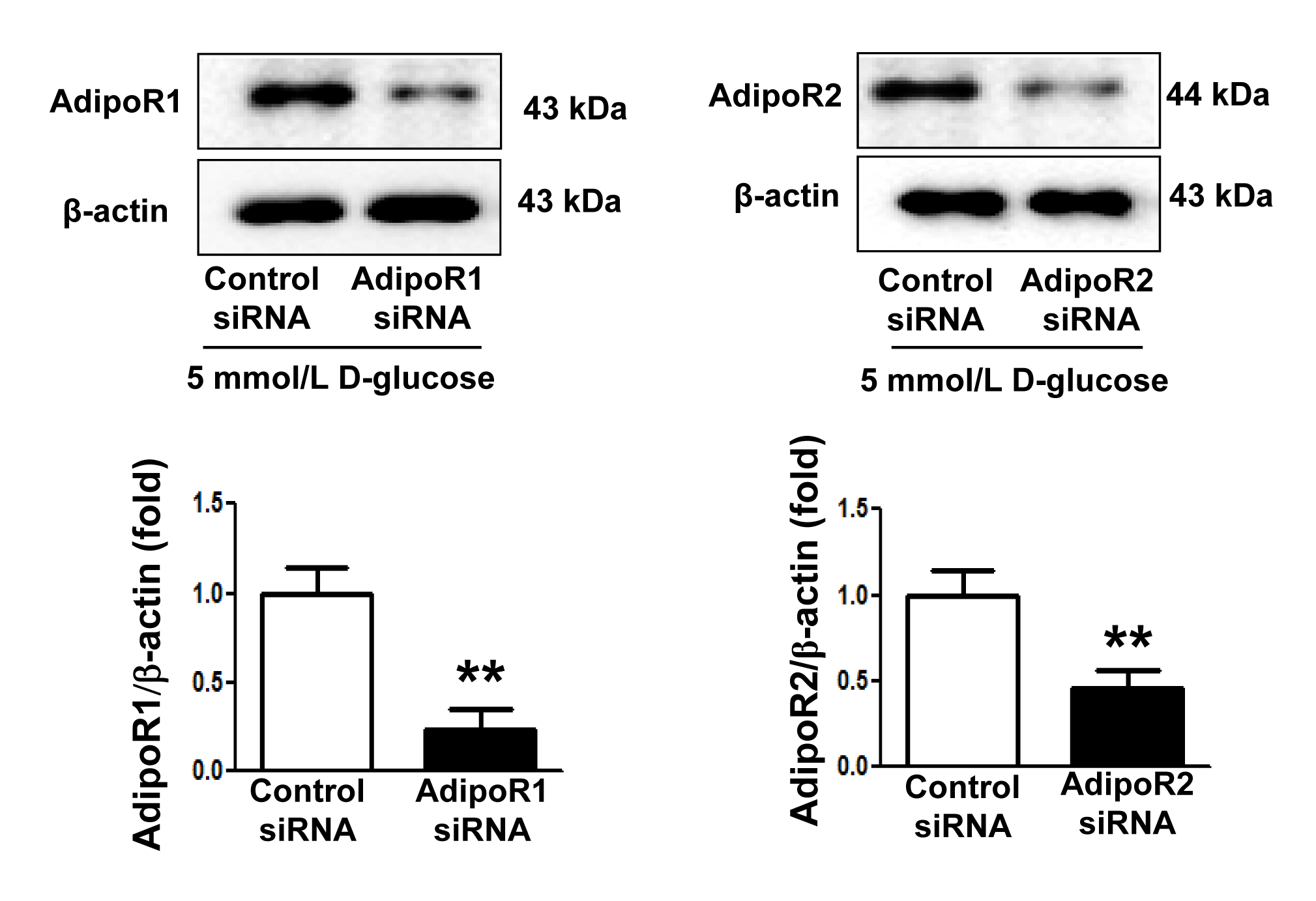

Supplement: Supplementary file 2 — 10.1186/s12967-016-0922-9 Downregulation of AdipoR1 and AdipoR2 expression in HGECs by siRNA. Levels of AdipoR1 and AdipoR2 expression were measured by Western blot. **p < 0.01 compared with control (scrambled) siRNA. [file 12967_2016_922_MOESM2_ESM.tif]
